# Correlation of CLDN18.2 and Tumor Microenvironment in Gastric Cancer: A Systematic Review

**DOI:** 10.3390/cancers17132120

**Published:** 2025-06-24

**Authors:** Katerina Zarampouka, Christos Tsiantas, Maria Athanasia Stavropoulou, Konstantinos Efthymiadis, Paschalis Theotokis, Sofia Gargani, Eleni Vrettou, Triantafyllia Koletsa, Maria Eleni Manthou, Soultana Meditskou

**Affiliations:** 1 Department of Pathology, Faculty of Medicine, Aristotle University of Thessaloniki, 54124 Thessaloniki, Greece; katerinazarampouka@hotmail.com (K.Z.); efthymiadis@gmail.com (K.E.); tkoletsa@auth.gr (T.K.); 2 Laboratory of Histology-Embryology, Department of Medicine, Faculty of Health Sciences, Aristotle University of Thessaloniki, 54124 Thessaloniki, Greece; ctsiant@auth.gr (C.T.); marathstav@gmail.com (M.A.S.); ptheotokis@auth.gr (P.T.); sgargani@bio.auth.gr (S.G.); evrettou@auth.gr (E.V.); mmanthou@auth.gr (M.E.M.)

**Keywords:** gastric cancer, CLDN18.2, tumor microenvironment

## Abstract

**Background/Objectives**: Gastric cancer (GC) is the fifth leading cause of cancer-related mortality. CLDN18.2 is a tight junction protein, expressed in gastric mucosa and is considered as a novel therapeutic target. Even though CLDN18.2 is associated with various components of the tumor microenvironment and the relation with clinical histopathological parameters has been widely studied, there is no sufficient data on the associations of CLDN18.2 expression and the components of the tumor microenvironment. This systematic review aims to gather and present all available data about the correlation of CLDN 18.2 expression and the tumor microenvironment. **Methods**: The research questions were systematically formulated using the PICO model to ensure clarity and precision, and the PRISMA flow diagram was constructed to detail the study selection process. **Results**: Sixteen original articles were retrieved. The major finding of this study was the positive correlation between CLDN18.2 expression and CD8+ T cells, neutrophils and cancer-associated fibroblasts. No correlation was found between CLDN18.2 expression and Tregs and B cells. For the remaining components of the microenvironment, there are contradictory data about their correlation with the expression of CLDN18.2. **Conclusions**: The tumor microenvironment plays a critical role in cancer progression and needs to be studied more thoroughly.

## Simple Summary

This systematic review aimed to explore the relationship between CLDN18.2 and the tumor microenvironment in gastric cancer. To achieve this, a thorough search of existing articles was performed across four scientific databases, using specific keywords related to gastric and gastroesophageal cancer and CLDN18.2. After applying exclusion criteria and removing duplicate entries, the remaining articles were carefully evaluated for relevance. Key data from the 16 included articles were organized into tables and summarized for analysis. The major finding of this study was the positive correlation between CLDN18.2 expression and CD8+ T cells, neutrophils, and cancer-associated fibroblasts. No correlation was found between CLDN 18.2 expression and Tregs and B cells. For the remaining components of the microenvironment, there are contradictory data about their correlation with the expression of CLDN18.2.

## 1. Introduction

Gastric cancer is the fifth most frequently diagnosed cancer globally and the fifth leading cause of cancer-related mortality. Global incidence varies significantly across different continents, with observed incidence rates exceeding 70% in Asia, with rates of 14% and 3% recorded for Europe and Northern America, respectively [1,2]. GC is characterized by poor survival outcomes, with 5-year overall survival dropping from 56.7% in stage I disease down to 5% in stage IV disease [3]. Most patients with GC are diagnosed with advanced stage disease after they become symptomatic, resulting in poor prognosis due to the limited efficacy of first-line therapies [4].

Chemotherapy with combination regimens has traditionally been the cornerstone in the treatment of advanced HER2-negative, locally advanced (LA) unresectable or metastatic gastric or gastro-esophageal junction (GEJ) adenocarcinoma [5].

In the area of immunotherapy, combinations of anti-PD1 immune checkpoint inhibitors (Nivolumab, Pembrolizumab, Tislelizumab) with first-line chemotherapy showed significant and clinically meaningful improvement in overall survival in patients with tumors expressing PD-L1 [6,7,8].

Numerous efforts to develop targeted therapies for the treatment of GC and GEJ tumors harboring specific molecular features have been futile in the past. Various targeted agents, such as EGFR targeting monoclonal antibodies Cetuximab [9] and Panitumumab [10] and anti-VEGFA monoclonal antibody Bevacizumab [11], failed to show survival benefits, when they were evaluated in combination with chemotherapy.

On the contrary, targeting HER2 expressing GC tumors has been successful. The addition of the anti-HER2 antibody Trastuzumab to chemotherapy, with or without Pembrolizumab depending on PD-L1 expression, has been established as the optimal treatment in the first-line setting for this subset of patients, offering a significant survival benefit compared to chemotherapy [9,12,13,14,15,16]. Therefore, there is a dire need for the identification and characterization of novel molecules that can be exploited for targeted treatment. Over the past decade, claudins have emerged as promising biomarkers for tailored therapy with targeted pharmaceutical agents to enhance survival rates in patients with advanced GC [4,17,18,19].

Claudin and occludin proteins are the two major components of tight junctions which bind to the PDZ domains of zonula occludens (ZO) proteins to anchor the actin cytoskeleton and establish the paracellular barrier between epithelial cells [20,21]. Altered claudin expression in GC leads to tight adhesion impairment and irregular cell polarity and disrupts acid resistance in the stomach and signaling pathways, inevitably contributing to increased cell proliferation, invasion, and metastasis [16,22,23,24,25,26]

The protein family of claudins consists of 27 known members expressed in various tissues and having a wide range of expression patterns in cancer [27,28,29,30]. The *CLDN18* gene is located in the long arm q3 of the second chromosome in humans; its estimated length is 35 kbp, and it weighs approximately 20–27 kDa [31] and is characterized by 6 exons and 5 introns [32]. It has two isoforms originating through alternative splicing of exon 1, *CLDN18.1* and *CDLN18.2*, with the former predominantly expressed in alveolar epithelial cells, and the latter explicitly expressed in gastric mucosa [31,33,34].

Because of CLDN18.2’s unique location at the apical region of the cell membrane, when normal epithelial cells are transformed to cancerous in gastric mucosa, CLDN18.2 epitopes are exposed due to changes in cell polarity, making claudin 18.2 a valuable target for antibody therapies [35,36,37,38]. Normally, this protein is expressed in paracellular spaces of gastric epithelia and is highly difficult to target, since targets directly associated with cancer development and expressed in the cell membrane are usually utilized in most targeted therapies. As a result, only when CLDN18.2 is exposed in malignant epithelia, can it be an accessible target for therapeutic interventions [39].

It is known that protein kinase C (PKC) or extracellular signal-related kinase (ERK) signaling pathways may contribute to ectopic expression [40,41]. Moreover, since it is not expressed in stem cells like other members of claudin family, the expected toxicity deriving from targeted therapies is highly reduced, making it a promising drug target [42].

Over the past decade, CLDN18.2 has emerged as a promising biomarker for tailored therapy with targeted pharmaceutical agents. The addition of monoclonal anti-CLDN18.2 antibody Zolbetuximab to chemotherapy has led to significant improvement in median OS and is now considered to be the standard-of-care option in the first-line setting for patients with CLDN18.2-positive, HER2-negative GC and GEJ tumors [43].

Moreover, ADCs targeting CLDN18.2, such as CMG901, are also evaluated in early-phase clinical trials [19]. It seems that Claudin 18.2 can be utilized for the development of monoclonal antibodies, bispecific antibodies, and cell-based therapies [44,45].

However, the role of CLDN18.2 in gastric cancer is complex and deeply interactive with the tumor microenvironment. This has been proved through GSEA analysis, where CLDN18.2 is highly associated with PD1 and Wnt pathways, regulation of B cell antigen receptor (BCR) signal transduction, immune modulation, interactions between cells in the vascular wall, and control of the cell cycle [46]. The tumor microenvironment is made up of the extracellular matrix and includes a variety of cells like cancer cells, cancer-associated fibroblasts (CAFs), pericytes, and immune cells [47,48]. While CLDN18.2’s expression in gastric and gastroesophageal cancer is widely correlated with clinical, immunohistopathological, and molecular features of tumor cells like HER2, MSI, PDL-1,2, EBV [49,50,51,52], there are few articles associating its expression with tumor microenvironment markers. BiTEs targeting both T cell CD3 and CLDN18.2, such as ASP2138, have currently been developed and show activity in pre-clinical cancer models [43]. Furthermore, treatment with CLDN18.2-specific CAR-T cells has also demonstrated therapeutic potential in pre-treated patients with CLDN18.2-positive advanced gastrointestinal cancer, including GC and GEJ cancer [44].

These recent surges in the development of targeted therapies against CLDN18.2 warrant further study of CLDN18.2 expression and its relation to cells and molecules of the tumor microenvironment to identify groups of patients achieving better disease responses. In this review, we aim to systematically present the relative results in accordance with the tumor’s microenvironment.

## 2. Materials and Methods

### 2.1. PICO Model

The PICO model was used to shape and define the research question for this study (Table 1). To ensure a thorough and systematic approach, the team followed the Preferred Reporting Items for Systematic Reviews and Meta-Analyses (PRISMA) guidelines when extracting, screening, and evaluating the relevant articles. Two pathologists independently assessed the articles for eligibility. In cases of disagreement, a third pathologist adjudicated the discrepancies, blinded to the identity of the initial reviewers’ decisions. This study protocol is registered in the OSF database (registration number: OSF | Correlation of CLDN18.2 and Tumor Microenvironment components).

### 2.2. PRISMA

**Figure 1:**
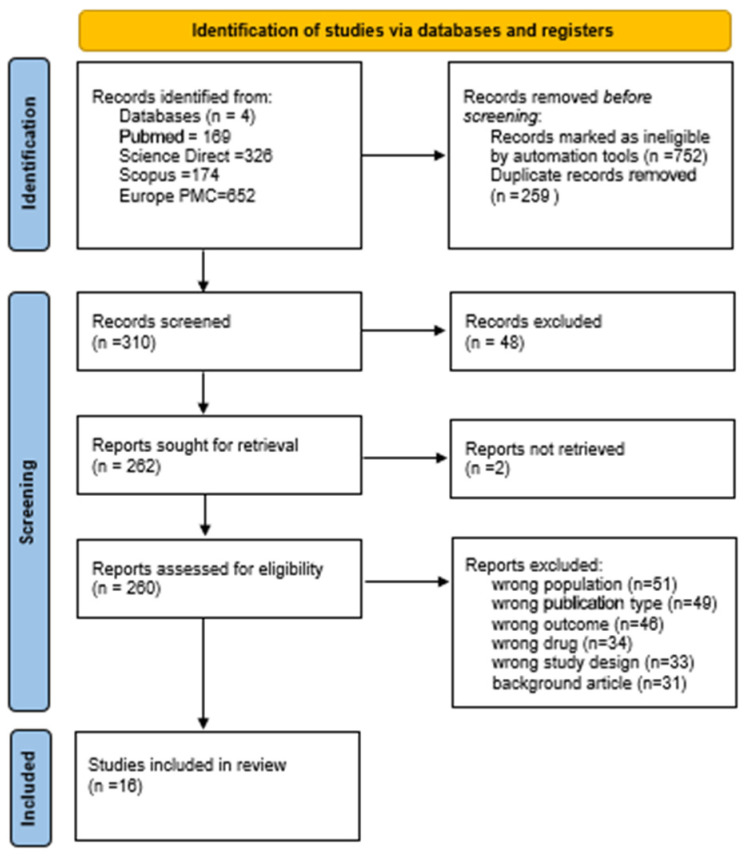
PRISMA flow diagram [53].

A PRISMA flow diagram was prepared using the code based on the keywords (((((gastric) OR (stomach)) OR (gastroesophageal)) AND (cancer*)) OR (carcinoma*)) OR (adenocarcinoma*)) AND (Claudin18.2)) OR (CLDN18.2) as illustrated in Figure 1. This systematic review included only original or research articles which referred to the tumor immune microenvironment and PD-L1 expressed by tumor cells. The articles had to be written in English. The databases used were PubMed, ScienceDirect, Scopus, and Europe PMC. Initially, the total number of all articles found without the inclusion criteria were recorded. Once the criteria were applied, the automation tools filtered out articles that were not relevant. The remaining articles were reviewed, with duplicates removed. Each article was carefully examined for its relevance by looking at the title, abstract, and main text.

## 3. Results

The infiltration of CD8+ T cells is positively correlated with CLDN18.2 expression in most published studies [46,54,55,56,57], except that of Kubota et al., 2023 [58]. Indeed, Jia et al. found positive correlation only for non-depleted CD8+ T cells (CD8+LAG-3-, CD8+PD-1-, CD8+TIM-3-, CD8+LAG-3-PD-1-, CD8+LAG-3-TIM-3-, CD8+PD-1-TIM-3-, CD8+LAG-3-PD-1-TIM-3-). TIM-3 (T cell Immunoglobulin and Mucin-Domain-3), LAG-3 (Lymphocyte Activation Gene 3) and PD-1 (Programmed cell death 1) are immune checkpoints which regulate the immune response [59] (Table 2). Low expression of these proteins is related to poor prognosis as is positive expression of CLDN18.2 [55].

Infiltration of CD4 T cells in the tumor core either is associated with positive expression of CLDN18.2 [55,56,57] or is not associated with CLDN expression [54]. In addition, lower expressions of CTLA-4 and PDL-1 in CD4 T cells are observed in CLDN18.2 positive group. Effector CD4 T cells were found positively correlated with CLDN18.2 expression [55]. A recent study by Kim et al. found that CLDN18.2 is positively correlated with the infiltration of CD8+ in the center of the tumor and the infiltration of CD4 in the periphery. The CD3 marker was found to be either positive or not correlated with CLDN18.2 expression [56].

Tregs (T regulatory cells) have not been correlated with CLDN18.2 in any existing studies [46,55]. Th1(T helper type 1 cells) and Th2 (T helper 2) have either been positive [57] or not correlated [46]. Tfh (T follicular helper cells) are included in one study, and they have not been correlated with CLDN18.2 expression [46]. On the other hand, Tγδ has been found to have a negative correlation with claudin expression according to Tao et al. [46].

All research teams found no correlation between B cells and claudin [46,54,55]. An exception to this was Wang et al. who found positive correlation in the TISIDB database [54].

Regarding macrophages, it is not clear if they have correlation with CLDN18.2 expression and with which type [46,55,57,58,60]. Monocytes are not correlated with CLDN18.2 expression [60]. NK cells (Natural Killer) were not correlated with CLDN18.2 expression in two studies with their own patients [60], and they were correlated negatively [49,60] in the studies which used databases. Neutrophils, according to the available sources, are positively correlated with CLDN18.2 expression [46,55]. DCs (Dendritic cells) were found only by Tao et al., and they may be positively correlated with CLDN18.2 [46]. Eosinophils, MDSCs, and NKT cells are negatively correlated with CLDN18.2 expression [46,57]. CAFs (cancer-associated fibroblasts) are positively correlated with the positive CLDN18.2 expression with great certainty (*p* < 0.001) [61].

Most of the studies indicate no correlation between CLDN18.2 expression and PD-L1 expression. On the other hand, Wang et al. (2023) and Matsuishi et al. (2024) found positive association of PD-L1 expression [54,60] and CLDN18.2 in contrast to Qi et al. (2024), who found 10% lower expression of PD-L1 in the CLDN18.2 (+) group independent of CPS status [64]. All in all, the existing evidence suggests a mostly positive correlation between CLDN18.2 expression and the presence of certain immune cells, such as CD8+ T cells, CD4+ effector T cells, neutrophils, and cancer-associated fibroblasts, whereas correlations with other cell types like macrophages and NK cells, as well as the PD-L1 expression, remain inconsistent and require further clarification.

## 4. Discussion

CLDN18.2, a member of the family of claudins that forms tight junctions, emerged as a new therapeutic target in gastric cancer in recent years. Overexpression of CLDN18.2 is often observed in gastric cancer, making it an attractive candidate for targeted therapies. Even though the correlation of CLDN18.2 expression and different clinicohistopathological parameters is widely studied, limited data exist regarding associations of CLDN18.2 expression and components of the existing microenvironment. In this review, we aim to systematically present the relative results in accordance with the tumor microenvironment.

What is important after identifying the correlation between the tumor microenvironment components and CLDN18.2 expression is the impact of that correlation on overall survival (OS). T lymphocyte subsets—including CD4+ helper T cells, regulatory T cells (Tregs), and CD8 + cytotoxic T cells—exert distinct immunological functions within the tumor microenvironment (TME). Among these, CD8+ T cells are primarily responsible for mediating cytotoxic antitumor activity. In gastric cancer (GC), the composition and intratumoral density of T cell subsets are significantly associated with clinical outcomes and therapeutic response [67]. Many studies in gastric cancer, independent of those focusing on claudins, have reported improved OS in cases with higher CD8+ T cell infiltration [68]. According to Kubota et al., CLDN18.2 expression is not correlated with OS, which may be attributed to the lack of association between CLDN18.2 expression and CD8 [58]. Wang et al. confirmed a positive correlation between OS and both CD8+ and CD4+ T cells. Even though they found a positive association between CLDN18.2 and CD8, in this publication, CLDN18.2 is recognized as an independent risk factor due to the outcomes of univariate and multivariate Cox analysis. For this reason, Wang et al. report that the subgroup with improved OS was characterized by CLDN18.2 negativity and high infiltration of CD4+ and CD8+ T cells [54].

In the research of Jia et al., a similar positive correlation between CD8+ T cells and CLDN18.2 expression was found, as well as poorer OS. Jia et al. did not indicate CLDN18.2 as an independent factor of overall survival and investigated the contradiction of high CD8+ T cells resulting in prolonged OS and the CLDN18.2-positive group having negative impact on OS. This is explained by Jia et al. with the analysis of biomarkers expressed in CD8 T cells. The analysis shows low expression of three immunoregulatory biomarkers, PD-1, LAG-3 and TIM-3 [59,69], which suggests reduced antitumor capabilities of cells. This reduced expression suggests impaired immune checkpoint signaling, potentially leading to inadequate T cell activation and compromised immune surveillance against tumors [55].

The microenvironment could help in the prediction of responses to different therapies. It is worth mentioning that CD3 was found to be either positively correlated or not correlated with CLDN18.2. This marker is crucial, due to the development of BiTes targeting CLDN18.2 and CD3 such as ASP2138, in pre-clinical cancer models [17,70]. Generally, no correlation of PD-L1 expression with CLDN18.2 indicates that anti PD-1/PD-L1 therapy will not be beneficial for patients with CLDN18.2-positive tumors. Also, neutrophils are positively correlated with CLDN18.2, and they are associated with poor therapeutic response and poor prognosis [71,72].

According to a recent meta-analysis, positive expression of CLDN18.2 is correlated with shorter OS [73]. From this aspect, T regs are unexpected not to be correlated with CLDN18.2 expression, because they have a role in tumor progression and contribute to immunosuppression [74,75,76]. Natural Killer cells were not found to be correlated in two IHC-based studies, while the two database-based studies reported conflicting results. Knowing that the heterogeneity of data in the databases is a crucial factor of bias and the other two studies have enough patients, the case of no correlation with CLDN18.2 is more reliable. No correlation between NK cells and CLDN18.2 is consistent with the result of the meta-analysis mentioned previously and the role of NK cells in antitumor immunity [77,78]. Regarding the relation between CLDN18.2 and CAFs, Liu et al. declares that CLDN18.2 promotes interaction between cancer cells and CAFs, taking on a more aggressive role that relies on CAF involvement. There is a crosstalk between cancer cells and CAFs via CLDN18.2 and S100A4 accordingly. So, combining CLDN18.2-targeted therapies with S100A4 inhibitors (or other CAF-targeted treatments) could be a therapeutic target by disrupting their interaction [61]. Even though we could reach a conclusion about the pattern of correlation between immune cells with CLDN18.2, we are still far away from understanding the interactions between those and the possible clinical impact.

Apart from the uncertainty in the actual relation of macrophages and CLDN18.2 expression, it is not clear whether an increased or decreased proportion of macrophages is desirable due to the complex supportive and inhibitory role of TAMs in tumor progression. M1 macrophages which produce type I proinflammatory cytokines such as IL-1β, IL-1α, IL-12, TNF-α, and GFAP seem to not be correlated with CLDN18.2 expression [79,80,81,82]. Conversely, “alternatively activated” M2 macrophages which produce type II cytokines [83,84] and have pro-tumorigenic functions may either be negative or not correlated with CLDN18.2 expression, highlighting a beneficial role for macrophages generally in the CLDN18.2-positive group, or positively correlated in case of a tumor-progression role of macrophages.

Our review has a few limitations. First, all the studies included are single-institution reports, with many employing retrospective analyses. This approach carries risks such as incomplete clinical information and potential biases in sample selection. Additionally, direct comparisons across studies are challenging due to variations in the biomarkers analyzed and differing cut-off points used to define CLDN18.2 positivity. It is worth mentioning that the calculation of H-score is not used by all authors, whereas others define a double cut-off value in immunostaining and percentage of stained tumor cells. So, these methodological differences complicate consistent interpretation of CLDN18.2 expression patterns. The inherent limitations of database mining studies compared to experimental IHC-based assessments further contribute to variability in findings. Lastly, accurately assessing CLDN18.2 expression remains difficult due to potential biases stemming from experimental procedures and subjective interpretations by pathologists. Potential publication bias should also be considered, as studies with non-significant findings may be under-represented in the literature.

## 5. Conclusions

A deeper understanding of CLDN18.2’s role within the gastric cancer tumor microenvironment (TME) is crucial for advancing targeted therapies. Though current data are limited, evidence suggests that CLDN18.2 expression may affect immune infiltration, immune evasion, and stromal dynamics. Large, standardized clinical studies are needed to elucidate these interactions by correlating CLDN18.2 expression with TME features. Such insights could inform the development of combination treatments targeting both CLDN18.2 and the TME.

## Figures and Tables

**Table 1:** PICO table outlining Population (P), Intervention (I), Comparator (C) and Outcome (O).

P	Patients with gastric cancer not receiving anti-CLDN18.2 therapy
I	Assessment of CLDN18.2 expression levels using immunohistochemistry (IHC), quantitative PCR, or other molecular techniques.
C	Tumor microenvironment components
O	Establish the correlation between CLDN18.2 expression and components of tumor microenvironment

IHC: immunohistochemistry.

**Table 2:** Summary of studies investigating the correlation of tumor microenvironment components with CLDN18.2 expression.

Study	Way of Assesment	Tumor Immune Microenvironment	Immune Checkpoints
Wang et al., 2023 [54]	IHC (H-score ≥ 6) TIME, TIMER database	↑ CD8+ T cells (*p* = 0.021); CD3 (*p* = 0.05), B cells (*p* < 0.001)	No correlation with Foxp3 Positive correlation with PD-L1
		CD4 T cells (*p* = 0.6), B cells (*p* = 0.112)	
Jia et al., 2022 [55]	IHC (≥2, ≥40%) TCGA database	↑ CD8+ T cells (*p* = 0.023), Non-depleted CD8+ T cells (*p* < 0.05), CD4 T cells (*p* = 0.045), effector CD4 T cells (*p* = 0.026), Neutrophils (*p* = 0.031)	No correlation with PD-L1
		- Depleted CD8 T cells (*p* = 0.71), Tregs (*p* = 0.47), B cells (*p* = 0.25), M1 (*p* = 0.5), M2 (*p* = 0.71)	
Kubota et al., 2023 [58]	IHC (>2, ≥75%)	↑ Macrophages (*p* = 0.037)	No correlation with PD-L1
		- CD8+ T cells (*p* = 0.808), CD56 (*p* = 0.789), CD3 (*p* = 0.457)	
		↓ CD16	
Matsuishi et al., 2024 [60]	IHC (>2, ≥75%)	NK cells (CD16, CD56, CD56dimCD16+, CD56brightCD16-), Monocytes (classical, intermediate, non-classical), Macrophages	No correlation with PD-L1(CPS: 1) Positive correlation with PD-L1(CPS: 5)
Liu et al., 2024 [61]		↑ CAFs (*p* < 0.01)	No
Tao et al., 2023 [46]	TCGA database	↑ CD8+ T cells (*p* < 0.01), Th17 (*p* < 0.01), aDC (*p* < 0.01), iDC (*p* < 0.001), Mast cells (*p* < 0.001), Neutrophils (*p* < 0.05).	No
		↓ Tγδ (*p* < 0.05), NK cells (*p* < 0.05), MDSC (*p* < 0.001)	
		- Tfh, Th1, Th2, Treg, B cells, Macrophages	
Ogawa et al., 2024 [62]	IHC (≥2, >40%)	No	No correlation with PD-L1
Pellino et al., 2021 [63]	IHC (>0, >0% and >2, >75%)	No	No correlation with PD-L1 (CPS: 1, CPS: 5)
Qi et al., 2024 [64]	IHC (>2, >40%)	No	Negative correlation with PD-L1 (CPS: 1, CPS: 5, CPS: 10)
Waters et al., 2024 [65]	IHC (>2, >50% and >75%)	No	No correlation with PD-L1(CPS > 1)
Kwak et al., 2025 [66]	IHC (>2, >75%)	No	Positive correlation with PD-L1 (CPS: 5) No correlation with PD-L1 (CPS: 1, CPS: 10)
Wu et al., 2024 [57]	AuCell (Xcell markers)	↓ NK, CD4+ T cells, Th2 cells, CD8+ T cells, CD4+ memory T cells, Th1 cells	No
		↑ Macrophages, NKT, Macrophages M2, Eosinophils	
Kim et al., 2025 [56]	IHC (>2, >75%)	↑ CD8+ T cells (center) (*p* = 0.041), CD4 T cells (periphery) (*p* = 0.04), CD3 (periphery) (*p* = 0.009)	No correlation with PD-L1 (CPS: 1, CPS: 5, CPS: 10)
		- CD8+ T cells (periphery) (*p* = 0.329), Foxp3 cells (center (*p* = 0.158) or periphery (*p* = 0.950)), CD4 (center) (*p* = 0.202) CD3 (center) (*p* = 0.140)	

↑, positive correlation; ↓, negative correlation; -, no correlation; No, no data available.

## Abbreviations

ADC (antibody drug conjugate); BiTE (bispecific T cell engager); CAFs (cancer-associated fibroblasts); CAR (chimeric antigen receptor); CLDN18.2 (claudin-18.2); CPS (combined positive score); DC (dendritic cell); EGFR (epidermal growth factor receptor); FLOT (5-fluorouracil, leucovorin, oxaliplatin, docetaxel); FOLFOX (5-fluorouracil, leucovorin, oxaliplatin); GC (gastric cancer); GEJ (gastroesophageal junction); GFAP (glial fibrillary acidic protein); HER2 (epidermal growth factor 2); HR (hazard ratio); H-score (histochemical score); ICI (immune checkpoint inhibitor); IHC (immunohistochemistry); IL-1α (interleukin-1 alpha); IL-12 (interleukin-12); LAG-3 (lymphocyte-activation gene 3); MDSC (myeloid-derived suppressor cell); MSI (microsatellite instability); NK (natural killer cell); NKT cells (natural killer T cells); OS (overall survival); PD-1 (programmed cell death protein 1); PD-L1 (programmed death-ligand 1); PICO (Population Intervention Comparator Outcome); PRISMA (Preferred Reporting Items for Systematic Reviews and Meta-Analyses); Th1 (T helper type 1 cells); Th2 (T helper type 2 cells); Tfh (T follicular helper cells); TIMER database (Tumor Immune Estimation Resource database); TCGA (The Cancer Genome Atlas); TIM-3 (T cell immunoglobulin and mucin-domain containing-3); TME (tumor microenvironment); Tregs (regulatory T cells); TSIDB database (Tumor–Stromal Interaction Database); TNF-α (tumor necrosis factor-alpha); VEGFA (vascular endothelial growth factor-A); Wnt (Wingless-related integration site); ZO (zonula occludens); PDZ (post synaptic density protein, Drosophila disc large tumor suppressor, zonula occludens-1 protein).
